# A Generalized National Planning Approach for Admission Capacity in Higher Education: A Nonlinear Integer Goal Programming Model with a Novel Differential Evolution Algorithm

**DOI:** 10.1155/2016/5207362

**Published:** 2015-12-24

**Authors:** Said Ali El-Qulity, Ali Wagdy Mohamed

**Affiliations:** ^1^Department of Industrial Engineering, Faculty of Engineering, King Abdulaziz University, P.O. Box 80200, Jeddah 21589, Saudi Arabia; ^2^Department of Operations Research, Institute of Statistical Studies and Research, Cairo University, Giza 12613, Egypt

## Abstract

This paper proposes a nonlinear integer goal programming model (NIGPM) for solving the general problem of admission capacity planning in a country as a whole. The work aims to satisfy most of the required key objectives of a country related to the enrollment problem for higher education. The system general outlines are developed along with the solution methodology for application to the time horizon in a given plan. The up-to-date data for Saudi Arabia is used as a case study and a novel evolutionary algorithm based on modified differential evolution (DE) algorithm is used to solve the complexity of the NIGPM generated for different goal priorities. The experimental results presented in this paper show their effectiveness in solving the admission capacity for higher education in terms of final solution quality and robustness.

## 1. Introduction

One of the key transformations in global higher education (HE) is the rapid growth of the sector. Growth started in the last four or five decades of the 20th century and continues after the turn of the century. Worldwide, the number of students in higher education has increased from 98 million in 2000 to over 150 million in 2007, implying a growth of over 50% in less than ten years. Worldwide gross enrolment ratios (defined as the total enrolment in HE, regardless of age, expressed as a percentage of the eligible official school-age population corresponding to the same level of education in a given school year) in the same period show an increase from 19% to 26% [1]. According to Trow [2], HE systems move from elite through mass to universal HE. Elite systems are characterized by low enrolment rates (between 0 and 15%). In systems of mass HE, the main function of higher education is transmission of skills and preparation for a broader range of technical and economic elite roles. Access is a right for those with certain qualifications and enrolment rates vary between 16 and 50%. Finally, universal HE is characterized by enrolment rates larger than 50%. In these systems, access to HE is perceived as an obligation for the middle and upper classes and the function of HE is related to adaptation of “whole population” to rapid social and technological change.

A growing demand for higher education requires on the supply side balanced growth in staff, both academic and administrative, and in facilities and infrastructure. However, growth in the supply of HE often is hampered by competition on the labor market for qualified personnel. Ashcroft and Rayner [3] indicate that, particularly, graduates with higher degrees are also in demand by the private and government sector.

The situation is aggravated when, as often is the case, the income is not keeping pace with the growth in student numbers. Without sufficient investments in facilities and infrastructure, institutions are left with “inadequate resources for books and journals, equipment, computers, and telecommunications” [4]. Furthermore, lack of funding leads to an increase in student staff ratios creating situations in which “students literally are unable to find room in classes” [5]. Rising social aspirations and growing socioeconomic relevance lead also to demands for increased performance of High Education in interrelated areas for increase in the labor market relevance of education and the supply of a more diversified student specialty suitable for the market needs. However, numerous reports indicate mismatches between supply and demand of graduates [6]. Lack of responsiveness of education systems to new labor market demands will hinder the development since research, for example, indicates that, in countries with more engineering students, the economy grows faster than in countries with more lawyers [7].

The Ministry of Higher Education in the Kingdom of Saudi Arabia is keen on working out its strategic plans and ensuring their compatibility with the government's development plan. To this effect, the ministry has put in perspective a number of vital objectives in its ninth five-year plan and the horizon plan for higher education (AAFAQ, 2014) [8] while attempting to benefit from the international trends by attracting international experts in the field of higher education strategic planning (Ministry of Higher Education, 2010) [9]. The ministry launched an initiative to prepare a modern and a long-term plan for university education to meet challenges of high population growth rate, ever-increasing funding demands, labor market needs for highly qualified graduates and student-to-faculty ratio, and so forth [10].

The higher education authority in any country always raises the question of admission capacity for the higher education institutions and how it is able to respond to the various challenges it is facing. The tradition way for tackling such a problem is to design separate plans that align with the specific needs and behaviors of each individual university to cope with the available resources and capacity.

The current paper presents a more generalized mathematical model for higher education sector that can successfully meet the national social, economic, and cultural challenges that face higher education admission capacity problems over the coming years. The model is general that it can be applied for different countries and/or universities.

It is known that GP is an extension of linear programming involving an objective function with multiple objectives [11]. The traditional GP model can be easily solved by simplex method or by using many computerized software programs such as Microsoft Excel Solver add-on and the LINGO package [12]. However, it should be noted that there are many other types of GP models that may include large-scale and nonlinear relations; such models with large number of integer variables add a computational challenge and extra level of difficulty for solving using classical programming techniques. Consequently, using metaheuristic techniques as a substitute for traditional programming methods in order to solve hard GP problems is an open research area [13, 14]. Thus, due to the complexity of the proposed model, a novel evolutionary algorithm based on modified constrained differential evolution algorithm is developed to solve the proposed nonlinear integer GP model.

The paper is organized as follows.  Section 2 handled the literature review for the problem under study.  Section 3 explained the general outlines of the system including all the inputs together with the components of the mathematical model and its objectives.  Section 4 explains the solution methodology over the planning horizon.  Section 5 showed how the proposed NIGPM is developed to adapt general proposed goals for a country.  Section 6 utilized the proposed NIGPM for Saudi Arabia as a case. The solution of the model and discussions are given as well. The proposed differential evolution approach and problem solutions were explained in Section 7. The conclusions and points for future researches are summarized in Section 8.

## 2. Literature Review

Over the last four decades, a variety of optimization methods have been developed to solve university admissions planning system. Virtually, one of the most effective and powerful mathematical programming techniques to formulate and solve optimization problems, proposed in the literature, is goal programming (GP) technique. A considerable number of research studies have been proposed to optimize university admission planning problems by using goal programming technique alone or combined with other classical mathematical methods or intelligent optimization algorithms. In fact, many academicians and researchers have embraced this technique as an appropriate technique in such optimization problem. The main reason of using GP is its capability of simultaneously satisfying several conflicting goals with varying priorities relevant to the decision-making situation. GP approach was firstly proposed by Lee and Clayton [15], for an optimum allocation of resources in institutions of higher learning. The scope of this study was limited to the planning of one college within the university. Additionally, the planning horizon was also limited to one year. This model was based on actual operational data at the College of Business, Virginia Polytechnic Institute and State University, United States of America. Many types of constraints and variables regarding the academic staff only were taken into consideration in this study such as total number of academic staff, distribution of academic staff, and number of graduate research assistants. However, all other remaining issues regarding academic process at universities were excluded. Likewise, Schroeder [16] introduced a new approach for recourse planning in the universities based on GP. Data were gathered at the University of Minnesota, Minnesota State, United States of America, representing three large academic departments over a three-year period. The goals were faculty instruction loads, staff-to-faculty ratios, faculty distribution by rank, and teaching-assistant-to-faculty ratios. These specified goals are achieved as closely as possible, subject to constraints on the projected budget available in each year of the planning horizon and to faculty-flow constraints. The decision variables are the faculty, staff, and teaching-assistant levels in each of the several academic units over the planning horizon. The model was used for long-range budget planning and resource allocation. On the other hand, Lee and Moore [17] developed a goal programming model to determine the basic composition of the total group of new students to be admitted into an educational institution. The data were obtained from a land-grant university; it is located in a southeastern state, United States of America. It determines the number of students that should be admitted in each of the various categories but does not specify which individual students should be offered admission. They proposed a very simple admissions planning model. The decision variables are defined as the number of admitted students in each category of in-state or out-of-state students; freshman, transfer, or readmitted students; and men or women students and all their possible combinations. Thus, they focused on formulating an admission policy for the newly entering students only. In 1981, Kendall and Luebbe [18] developed a GP model to manage recruitment activities in the small four-year Concordia College in Nebraska. Their model identifies the type and number of activities that must be completed each quarter in order to reach an enrollment goal for a given year. These activities included budget, time, manpower, and marketing strategies. The goal was to enable recruiters to meet enrollments while managing recruiting resources and activities in order to remain within the recruiting budget. They concentrated on university financial related problems for private colleges. Soyibo and Lee [19] developed a large-scale GP model for efficient resource allocation for Ibadan University, Nigeria, which includes eight faculties and a college of medicine over a five-year planning horizon. This model defines student enrollment and academic staff level goals. Linear programming techniques have been also used in higher education planning. In 2009, Khan [20] used a product mix model of linear programming for university's optimal enrollment management. The aim is to have the best tuition contribution to the campus using the best student mix and optimal use of those constraints that impact student enrollment every semester. In 2013, Kassa [21] used a linear programming approach for placement of applicants to study programs developed and implemented at the College of Business & Economics, Bahir Dar University, in Ethiopia. The approach is estimated to significantly streamline the placement decision process at the college by reducing required man-hour as well as the time it takes to announce placement decisions. Decision support system has been proposed as a new trend of research in higher education in university planning system as the academic financial planning, admission policy, resource allocation, recruiting, managing university fund, budgeting, and classroom scheduling have become a highly complex system with huge data bases. Therefore, several attempts in developing DSS to deal with one or more of these subsystems have been done. Resource allocation in a university received considerable attention from several authors. A goal programming-based DSS has been presented by Franz et al. [22]. In this approach, they attempted to adopt a variety of academic decision-makers, with differing planning views in an environment of multiple conflicting objectives. They report that testing of their DSS on four academic decision-makers in large US Midwestern University shows considerable promise for supporting decision-makers with varied problem-solving styles. Similarly, to find the optimal admission policy, a DSS for student admission policy to Kuwait University, Kuwait, has been developed by Eliman [23]. The DSS is composed of three modeling components: first, the academic performance analysis model which consists of two parts (a multiclassification analysis (MCA) and cohort analysis); second, models to estimate secondary school graduates supply and demand for university graduates using demographic growth and regression analysis; third, student allocation models that use a linear programming formulation. The overall reaction of the decision-makers in Kuwait University to the DSS has been positive. In the same context, Vinnik and Scholl [24] proposed UNICAP (acronym for university's capacity planning), which was aimed at optimizing the academic decision-making and admission capacity planning, by allowing simulation and evaluation of strategic plans. The system integrates data from heterogeneous sources, applies OLAP (online analytical processing) and data warehouse techniques, and allows users to interact with it in order to test various development strategies and become aware of their quantitative implications. The user interface of UNICAP is assured by providing orientation aids, detailed instructions, and graphical support and leading the user through the computation. Visually enhanced presentation of the output facilitates its perception and interpretation. Recently, multiaggregator models for fuzzy queries and ranking based on an evolutionary computing approach to build a decision support system for admission student in university have been introduced by Alsharafat [25]. A unified approach based on a combination of four soft computing methodologies (Fuzzy Logic, Neuronetworks, Genetic Algorithms, and Probabilistic Reasoning) was used to build the proposed intelligent DSS. The information provided in this study was a hypothetical situation that will reflect future admissions criteria. Based on the above literature review from different points of view to deal with this problem through years, it can be concluded that the resource planning of higher education in university is still an open research area and many further studies must be carried out in different directions to build an efficient, effective, and integrated decision support system able to solve the majority of subsystems of university resource planning by incorporating simulation-optimization computer programs and intelligent data processing techniques to answer what-if scenarios and determining the optimal one or, alternatively, develop an appropriate optimization methodology using mathematical methods coupling with soft computing techniques to handle one subsystem proficiently by taking into consideration all factors that affect it as presented in the proposed research work.

## 3. System General Outlines

As can be clearly seen from the previously studied literature review, a considerable number of research studies have been proposed to model university admission planning problems. Some of these studies used goal programming technique alone or combined with other classical mathematical methods or intelligent optimization algorithms.

The scope of all of these mentioned studies was limited to the admission planning related to one unique college or institution. Most of them are concerned with available capacity and resources. Additionally, the planning horizon was limited to one upcoming year. The tradition way used for tackling such a problem is to design a separate plan that aligns with the specific needs and behaviors of each individual institute to cope with the available resources and capacity. None of the mentioned studies tackled a global view and solve the comprehensive problem at a national level and solve for accomplishing the national objectives of a country with respect to admission problem in higher education taking into consideration the social needs and the various objectives stated in the national development plan.

The current paper presents a new more generalized mathematical model for higher education sector in two ways: application on the national level and the long-term planning horizon. A case study in the Kingdom of Saudi Arabia is presented to clarify the idea. Meanwhile, the proposed model is designed in a general way that it can be adopted to be suited for different countries and/or universities.

**Figure 1:**
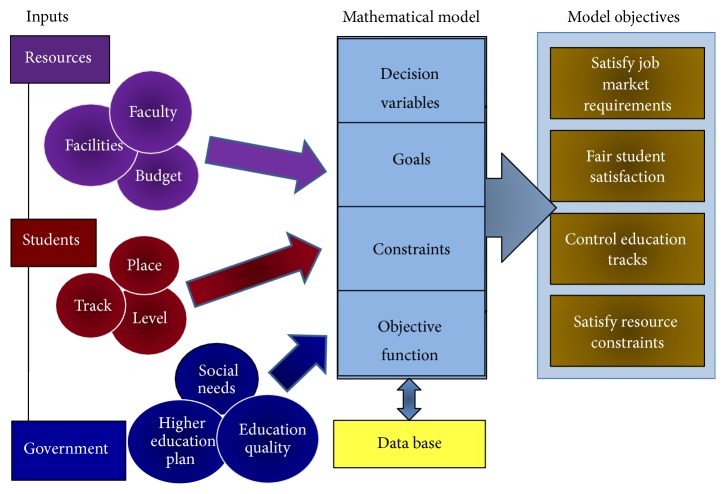
Admission system general outlines view.

Similar to higher education systems throughout much of the world higher education, Saudi Arabia faces a number of challenges. Among such challenges are the social needs expressed by the increasing demand for higher education and the correspondence between the admission capacity and the population, the available resources expressed by facilities, faculty, and budget, student needs expressed by location and education track limited by student levels, and the job market needs.  Figure 1 demonstrates all the mentioned factors as inputs to the proposed admission system outlines together with the components of the mathematical model and its objectives.

The proposed goal programming model will consider all the above inputs; then it will design the required unknown decision variables, goals, constraints, and objective function to satisfy the long-term plan that would help face up the various challenges standing in the way of all the higher educational institutions.

The main objectives for the model are to cope with the increasing demand for higher education in the country and to satisfy the job market requirements, fair student satisfaction, and control over the education tracks (medical, science and engineering, and arts) under the limitation of available resources.

## 4. Solution Methodology

**Figure 2:**
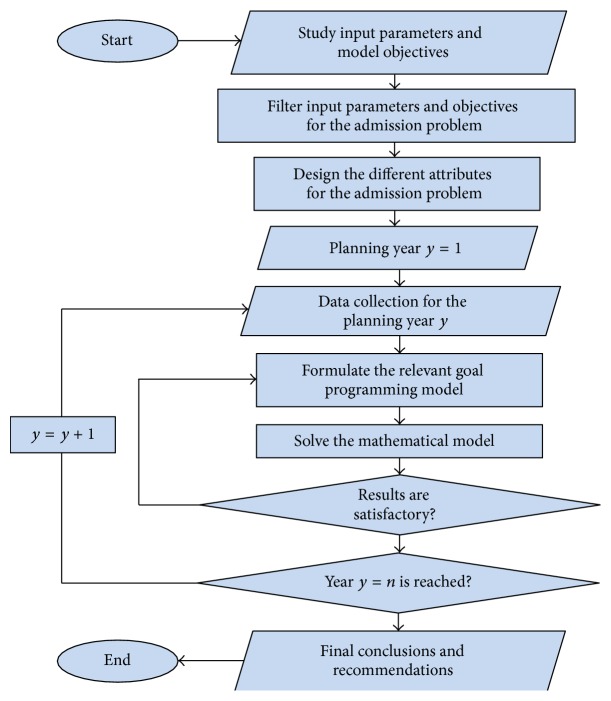
Flowchart of the general solution methodology.

The algorithm of solution starts with studying the strategic plans in the country related to higher education to extract the objectives intended for the admission problem.  Figure 2 represents the complete steps to solve the problem for different years of the planning horizon, *n*. Since each year has its specific data, the mode will be formulated and solved initially for the first year. The obtained results will be given as some of the input data for the second year, and so on, till reaching the last year of the plan.

## 5. Goal Programming Model for the Admission Problem

Firms often have more than one goal; they may want to achieve several, sometimes contradictory, goals. It is not always possible to satisfy every goal so goal programming attempts to reach a satisfactory level of multiple objectives.

The main difference is in the objective function where goal programming tries to minimize the deviations between goals and what we can actually achieve within the given constraints. The mathematical model will cover the main objectives stated in the current KSA Development Plan and that stated in KSA Higher Education Strategic Plan (AAFAQ) for the next 25 years. It will be restricted also to the budget and staff constraints as problem resources.

### 5.1. Decision Variables

To design the decision variables for the problem, it is necessary to represent all the different problem attributes. Let the decision variables be denoted by *x*_*i*,*j*,*k*_^*y*,*u*^ = *x*_status,  gender,  program_^year  of  the  plan,university^ = number of students, where different attributes are shown in Table 1.

### 5.2. Problem Goals

The Kingdom Development Plan and AAFAQ Project objectives are formulated to represent the mathematical model goals as follows.

Let

 
*d*_*n*_^*y*−^ be underachievement of the *n*th target in year *y*,
 
*d*_*n*_^*y*+^ be overachievement of the *n*th target in year *y*,

where *n* is the number of the constraints.

The deviation variables that need to be minimized will be included in the objective function and in the corresponding constraint, but those whose values are permitted to have nonzero positive values will be omitted from both the objective function and the corresponding constraints. The constraints are adjusted correspondingly to be of equality or nonequality types.

#### 5.2.1. Increase in the Enrollment Rate

It is required to increase the enrollment of students in higher education with an average annual growth rate of *p*_*r*_^*y*^:

(1) ∑u∈U∑k∈KxE,b,ky,u−∑u∈U∑k∈KxE,b,ky−1,u∑u∈U∑k∈KxE,b,ky−1,u+d1y−≥pry∑u∈U∑k∈KxE,g,ky,u−∑u∈U∑k∈KxE,g,ky−1,u∑u∈U∑k∈KxE,g,ky−1,u+d2y−≥pryy=1,2,3,4,5.

#### 5.2.2. Control the Education Tracks

The percentage of the total number of students enrolled in science and technology programs to the total number of students enrolled in higher education = *p*_*s*_^*y*^ and the percentage of the total number of students enrolled in medical programs to the total number of students enrolled in science and engineering = *p*_*m*_^*y*^ are as follows:

(2) ∑u∈U∑k=m,sxE,b,ky,u∑u∈U∑j∈JxE,b,jy−1,u+d3y−−d3y+=psy,∑u∈U∑k=m,sxE,g,ky,u∑u∈U∑k∈KxE,g,ky−1,u+d4y−−d4y+=psy,∑u∈U∑k=m∈MxE,b,ky,u∑u∈U∑k=s∈SxE,b,jy−1,u+d5y−−d5y+=pmy,∑u∈U∑k=m∈MxE,g,ky,u∑u∈U∑k=s∈SxE,g,ky−1,u+d6y−−d6y+=pmy,y=1,2,3,4,5.

#### 5.2.3. Percentage of Enrollment to Population and Percentage of Enrollment to High School Graduates

The percentage of total enrollment in higher education, regardless of age, to the total population in the age group of 18–23 years ≥*p*_*p*_^*y*^ and the accepted percentage in higher education from high school graduates in the same year, *p*_*h*_^*y*^, are as follows:

(3) ∑u∈U ∑j∈J ∑k∈KxE,j,ky,u+d7y−≥max⁡15·ppy·NCy−1,phy·NHy−1,y=1,2,3,4,5,

where *p*_*p*_^*y*^ is the percentage of total enrollment in higher education, regardless of age, to the total population in the age group of 18–23 years in year *y*. *N*_*C*_^*y*^ is the population of Saudi Arabia in the age of 18–23 years in year *y*. *p*_*h*_^*y*^ is the accepted percentage in higher education from high school graduates in year *y*. *N*_*H*_^*y*^ represents high school graduates in year *y* that will be decreased by the number of boys for bachelor scholarships abroad (*N*_*b*_^*y*−1^) and the number of girls for bachelor scholarships abroad (*N*_*g*_^*y*−1^).

#### 5.2.4. Enrollment and Student-to-Faculty Ratio

The percentages of the total number of students in each discipline of university education to the total faculty (*F*) in that specialty are as follows:

 medicine (*m* ∈ *M*) =  *β*_*M*_^*y*^,
 engineering and science (*e* ∈ *E*) = *β*_*E*_^*y*^,
 arts (*a* ∈ *A*) = *β*_*A*_^*y*^,
 total university (*u* ∈ *U*) = *β*_*U*_^*y*^:
  (4) ∑u∈U ∑m∈MxE,b,my,u−d8y+≤1tM·βMy·Fb,My,U,∑u∈U ∑m∈MxE,g,my,u−d9y+≤1tM·βMy·Fg,My,U,∑u∈U ∑s∈SxE,b,sy,u−d10y+≤1tS·βSy·Fb,Sy,U,∑u∈U ∑s∈SxE,g,sy,u−d11y+≤1tS·βSy·Fg,My,U,∑u∈U ∑a∈AxE,b,ay,u−d12y+≤1tA·βAy·Fb,Ay,U,∑u∈U ∑a∈AxE,g,ay,u−d13y+≤1tA·βAy·Fg,Ay,U,∑u∈U ∑k∈KxE,b,ky,u−d14y+≤1tM·βUy·Fb,Ty,U,∑u∈U ∑k∈KxE,g,ky,u−d15y+≤1tM·βUy·Fg,Ty,U,y=1,2,3,4,5,

where *F*_*j*,*K*_^*y*,*U*^ is the number of faculty members for gender *j* in specialty *K* in all universities *U*; *j* = *b*, *g*; *K* = *M*, *S*, *A* and *U*; *t*_*k*_ is the number of years in a program *k*.

#### 5.2.5. Resources Constraints for Enrollment

All the resources of the teaching process are collected in the total budget required for a university *u* that should not exceed a certain total limit of *B*_*u*_^*y*^ at any year *y* of the planning horizon.

Let

 
*c*_*u*_^*y*,*u*^ be the cost per student in a university *u* in a year *y*,
 
*B*_*u*_^*y*^ be the maximum budget for a university *u* in a year *y*, *y* = 1,2, 3,4, 5.

Then,

(5) $$\begin{array}{c} \underset{u\in U}{\sum }\;\underset{j\in J}{\sum }\;\underset{k\in K}{\sum }x_{E,j,k}^{y,u}-d_{16}^{y+}\leq \underset{u\in U}{\sum }\frac{B_{u}^{y}}{c_{u}^{y,u}},\;y=1,2,3,4,5. \end{array}$$

#### 5.2.6. Student Fair Satisfaction

The student fair satisfaction with respect to the geographical location for enrollment in the nearest university to his/her town and the education track he/she prefers will be fairly accomplished for all the students according to the available places and the preference parameters such as GPA, home town and marks in different courses, and the student's prioritized desires.

#### 5.2.7. Logic Constraints

The total number of students in all the universities in any year *y* is equal to the total number of students in all the specialties: medical, science and engineering, and arts; this is applied for both boys and girls as follows:

(6) ∑u∈U ∑k∈KxE,b,ky,u=∑u∈U ∑k=mxE,b,ky,u+∑u∈U ∑k=sxE,b,ky,u+∑u∈U ∑k=axE,b,ky,u,∑u∈U ∑k∈KxE,g,ky,u=∑u∈U ∑k=mxE,g,ky,u+∑u∈U ∑k=sxE,g,ky,u+∑u∈U ∑k=axE,g,ky,uy=1,2,3,4,5.

#### 5.2.8. Increase in the Graduated Students

With the plan projects over the same period, the number of graduates will increase with an average annual rate of *q*_*r*_^*y*^.

#### 5.2.9. Increase in the Graduation Rate

The percentage of students who have completed their studies in a given year to the total number of students enrolled in universities five years before that year is *q*_*d*_^*y*^.

These two goals will be expressed as follows:

(7) ∑u∈U ∑m∈MxG,b,my,u+d17y−≥max⁡1+qry·∑u∈U ∑m∈MxG,b,my−1,u,qdy·∑u∈U ∑m∈MxE,b,my−5,u,∑u∈U ∑s∈SxG,b,sy,u+d18y−≥max⁡1+qry·∑u∈U ∑s∈SxG,b,sy−1,u,qdy·∑u∈U ∑s∈SxE,b,sy−5,u,∑u∈U ∑a∈AxG,b,ay,u+d19y−≥max⁡1+qry·∑u∈U ∑a∈AxG,b,ay−1,u,qdy·∑u∈U ∑a∈AxE,b,ay−5,u,∑u∈U ∑m∈MxG,g,my,u+d20y−≥max⁡1+qry·∑u∈U ∑m∈MxG,g,my−1,u,qdy·∑u∈U ∑m∈MxE,g,my−5,u,∑u∈U ∑s∈SxG,g,sy,u+d21y−≥max⁡1+qry·∑u∈U ∑s∈SxG,g,sy−1,u,qdy·∑u∈U ∑s∈SxE,g,sy−5,u,∑u∈U ∑a∈AxG,g,ay,u+d22y−≥max⁡1+qry·∑u∈U ∑a∈AxG,g,ay−1,u,qdy·∑u∈U ∑a∈AxE,g,ay−5,uy=1,2,3,4,5.

#### 5.2.10. Job Market Requirements

Let *N*_*j*,*k*_^*y*^ be the number of available jobs in the country for gender *j* = *b* and *g* and specialty *k* = *m*, *s*, and *e*, in a year *y*; then,

(8) ∑u∈U ∑m∈MxG,b,my,u−d23y+≤Nb,my,∑u∈U ∑s∈SxG,b,sy,u−d24y+≤Nb,sy,∑u∈U ∑a∈AxG,b,ay,u−d25y+≤Nb,ay,∑u∈U ∑m∈MxG,g,my,u−d26y+≤Ng,my,∑u∈U ∑s∈SxG,g,sy,u−d27y+≤Ng,sy,∑u∈U ∑a∈AxG,g,ay,u−d28y+≤Ng,ay,y=1,2,3,4,5.

### 5.3. Objective Function

Once all goals and constraints are identified, management should analyze each goal to see whether underachievement or overachievement of that goal is an acceptable situation:

- If overachievement is acceptable, the appropriate corresponding deviation variable can be eliminated from the objective function.
- If underachievement is okay, the corresponding deviation variable should be dropped.
- If management seeks to attain a goal exactly, both deviation variables must appear in the objective function.

Typically, goals set by management can be achieved only at the expense of other goals. A hierarchy of importance needs to be established so that higher-priority goals are satisfied before lower-priority goals are addressed. Priorities (*P*_*i*_'s) are assigned to each deviational variable with the ranking so that *P*_1_ is the most important goal, *P*_2_ the next most important, *P*_3_ the third, and so on.

In our problem formulation, the goals and systems related to planning year 1 have the highest priority, and those related to planning year 2 are higher than those related to years 3, 4, and 5, and so on. The same weights will be given to all the goals in the same priority level. Accordingly, the problem will be divided into several problems; each one is related only to one planning year. The results obtained from each priority will be considered as constraints for the other priority levels.

So, the objective function is formulated as follows:

(9) Minimize:  z=d1y−+d2y−+d3y−+d3y++d4y−+d4y++d5y−+d5y++d6y−+d6y++d7y−+d8y++d9y++d10y++d11y++d12y++d13y++d14y++d15y++d16y++d17y−+d18y−+d19y−+d20y−+d21y−+d22y−+d23y++d24y++d25y++d26y++d27y++d28y+.

## 6. Kingdom of Saudi Arabia as a Case Study

Substituting *y* = 1 for the whole mathematical model, we will have the first priority level and the first part of the mathematical model. The following data are collected related to the Kingdom of Saudi Arabia; see Table 2.

It can be noticed that the mathematical model contains two distinct parts; one is related to the enrollment process while the other is related to the graduation process. The enrollment part is common for the first 18 goals, while the graduation part is specified in the remaining constraints while their related decision variables are not included in both the objective function and the enrollment constraints.

Constraints numbers 19–24 concerning the number of graduates can be completely satisfied without any effect on other parts of the enrollment process.

The problem of student enrollment is solved using the proposed differential evolution approach with data representing the first year of the National Plan for the Kingdom of Saudi Arabia.

## 7. The Proposed Differential Evolution Approach

It can be seen that the proposed GPM contains nonlinear constraints and involves a large amount of integer variables and it is not as simple as the linear GP model with continuous variables. Therefore, a novel constrained optimization based on modified differential evolution algorithm named COMDE (Mohamed and Sabry, 2012) [26] is used to solve the proposed nonlinear integer GP problem. Actually, the effectiveness and benefits of the new directed mutation strategy and modified basic strategy used in COMDE have been experimentally investigated. Numerical experiments on 13 well-known benchmark test functions and five engineering design problems have shown that the new approach is efficient, effective, and robust. The comparison results between the COMDE and the other twenty-eight state-of-the-art evolutionary algorithms indicate that the proposed COMDE algorithm is competitive with, and in some cases superior to, other existing algorithms in terms of the quality, efficiency, convergence rate, and robustness of the final solution. Thus, due its previous success, it is used here as an optimization approach with simple modification to handle integer variables, as will be mentioned in Section 7.3, without any modification to solve admission problems in higher education. Consequently, we use our algorithm to solve a real world problem which is similar to other benchmark problems in their mathematical features. Differential evolution (DE) has been receiving great attention and has also been successfully applied in many research fields in the last decade (Das and Suganthan, 2011) [27]. However, to the best of our knowledge, this is the first time to use DE in solving admission problems in higher education.

### 7.1. Differential Evolution (DE)

Differential evolution (DE) is a stochastic population-based search method, proposed by Das and Suganthan [27]. DE is relatively recent EAs for solving real-parameter optimization problems [28]. DE has many advantages including simplicity of implementation, reliability, and robustness and in general is considered as an effective global optimization algorithm [29]. In this paper, the scheme which can be classified using the notation as DE/rand/1/bin strategy is used [30, 31]. This strategy is the most often used in practice. A set of *D* optimization parameters is called an individual, which is represented by a *D*-dimensional parameter vector.

A population consists of NP parameter vectors *x*_*i*_^*G*^, *i* = 1,2,…, NP. *G* denotes one generation.

NP is the number of members in a population. It does not change during the process. The initial population is chosen randomly with uniform distribution in the search space. DE has three operators: mutation, crossover, and selection. The crucial idea behind DE is a scheme for generating trial vectors. Mutation and crossover operators are used to generate trial vectors, and the selection operator then determines which of the vectors will survive into the next generation [31–34].

#### 7.1.1. Initialization

In order to establish a starting point for the optimization process, an initial population must be created. Typically, each decision parameter in every vector of the initial population is assigned a randomly chosen value from the boundary constraints:

(10) $$\begin{array}{c} x_{ij}^{0}=l_{j}+rand_{j}*\left(\;u_{j}-l_{j}\;\right), \end{array}$$

where rand_*j*_ denotes a uniformly distributed number in the range [0, 1], generating a new value for each decision parameter. *l*_*i*_ and *u*_*i*_ are the lower and upper bounds for the *j*th decision parameter, respectively [28].

#### 7.1.2. Mutation

For each target vector *x*_*i*_^*G*^, a mutant vector *v* is generated according to the following:

(11) $$\begin{array}{c} v_{i}^{G+1}=x_{r_{1}}^{G}+F*\left(\;x_{r_{2}}^{G}-x_{r_{3}}^{G}\;\right),\;r_{1}\neq r_{2}\neq r_{3}\neq i, \end{array}$$

with randomly chosen indices and *r*_1_, *r*_2_, and *r*_3_ ∈ {1,2,…, NP}.

Note that these indices have to be different from each other and from the running index *i* so that NP must be at least four. *F* is a real number to control the amplification of the difference vector (*x*_*r*_2__^*G*^ − *x*_*r*_3__^*G*^). According to [29], the range of *F* is in [0, 2]. If a component of a mutant vector goes off the search space, that is, if a component of a mutant vector violates the boundary constraints, then the new value of this component is generated using (10).

#### 7.1.3. Crossover

The target vector is mixed with the mutated vector, using the following scheme, to yield the trial vector *u*:

(12) $$\begin{array}{c} u_{ij}^{G+1}=\left\{\begin{array}{cc} v_{ij}^{G+1}, & \mathrm{rand}\left(j\right)\leq CR\text{ or }j=\mathrm{rand}\;n\left(i\right),\\ x_{ij}^{G}, & \mathrm{rand}\left(j\right)>CR\;and\;j\neq \mathrm{rand}\;n\left(i\right), \end{array}\right. \end{array}$$

where *j* = 1,2,…, *D* and rand⁡(*j*) ∈ [0,1] is the *j*th evaluation of a uniform random generator number. CR ∈ [0,1] is the crossover probability constant, which has to be determined by the user. rand⁡*n*(*i*) ∈ {1,2,…, *D*} is a randomly chosen index which ensures that *u*_*i*_^*G*+1^ gets at least one element from *v*_*i*_^*G*+1^.

#### 7.1.4. Selection

DE adapts a greedy selection strategy. If and only if the trial vector *u*_*i*_^*G*+1^ yields a better fitness function value than *x*_*i*_^*G*^, then *u*_*i*_^*G*+1^ is set to *x*_*i*_^*G*+1^. Otherwise, the old value *x*_*i*_^*G*^ is retained. The selection scheme is as follows (for a minimization problem):

(13) $$\begin{array}{c} x_{i}^{G+1}=\left\{\begin{array}{cc} u_{i}^{G+1}, & f\left(u_{i}^{G+1}\right)<f\left(x_{i}^{G}\right),\\ x_{i}^{G}, & f\left(u_{i}^{G+1}\right)\geq f\left(x_{i}^{G}\right). \end{array}\right. \end{array}$$

A detailed description of standard DE algorithm is given in Algorithm 1.

rand⁡[0,1) is a function that returns a real number between 0 and 1. randint (min, max) is a function that returns an integer number between min and max. NP, GEN, CR, and *F* are user-defined parameters. *D* is the dimensionality of the problem.

### 7.2. Constrained Optimization Based on Modified Differential Evolution Algorithm (COMDE)

All evolutionary algorithms, including DE, are stochastic population-based search methods. Accordingly, there is no guarantee that the global optimal solution will be reached consistently. Furthermore, they are not originally designed to solve constrained optimization problems. Nonetheless, adjusting control parameters such as the scaling factor, the crossover rate, and the population size, alongside developing an appropriate mutation scheme and coupling with suitable and effective constraint-handling techniques, can considerably improve the search capability of DE algorithms. Therefore, in the proposed algorithm, a new directed mutation rule, based on the weighted difference vector between the best and the worst individuals at a particular generation, is introduced. The new directed mutation rule is combined with the modified basic mutation strategy DE/rand/1/bin, where only one of the two mutation rules is applied with the probability of 0.5. The proposed mutation rule is shown to enhance the local search ability of the basic differential evolution (DE) and to get a better tradeoff between convergence rate and robustness.

Two new scaling factors are introduced as uniform random variables to improve the diversity of the population and to bias the search direction. Additionally, a dynamic nonlinear increased crossover probability is utilized to balance the global exploration and local exploitation. COMDE also includes a modified constraint-handling technique based on feasibility and the sum of constraints violations. A new dynamic tolerance technique to handle equality constraints is also adopted. However, the test problem contains many equality constraints which is considered a very difficult problem. Thus, in order to increase the number of infeasible solutions to be improved through generations and become feasible with true feasible region, the initial tolerance *a* = 100, where *F*_initial_ = −log_10_(*a*), *F*_final_ = 4, and *R* = 0.75, the factor equation decreases linearly with *k* = 1. The required population size NP is 200 and max generation GEN = 2500. Readers are referred to [26] for details of the designed DE algorithm and its comparative results on benchmark test problems. The working procedure of the designed COMDE algorithm is presented in Algorithm 2. The parameter settings required for COMDE are shown in Table 3.

### 7.3. Handling of Integer Variables

In its canonical form, the differential evolution algorithm and COMDE algorithm are only capable of optimizing unconstrained problems with continuous variables. However, there are very few attempts to transform the canonical DE and proposed COMDE algorithms to handle integer variables [34–37]. In this research, only a couple of simple modifications are required: the new generation of initial population and boundary constraints verification, the proposed novel mutation operation, and the basic mutation schemes use rounding operator, where the operator round(*x*) rounds the elements of *x* to the nearest integers. Therefore, the initialization and mutations are as follows:

(i) Initialization and boundary constraint verification: *x*_*ij*_^0^ = round(*l*_*j*_ + rand_*j*_ *∗*(*u*_*j*_ − *l*_*j*_)).
(ii) New directed mutation: *u*_*ij*_^*G*+1^ = round(*x*_*r*1_^*G*^ + *F*_*l*_ *∗*(*x*_best_^*G*^ − *x*_worst_^*G*^)).
(iii) Basic mutation: *u*_*ij*_^*G*+1^ = round(*x*_*r*1_^*G*^ + *F*_*g*_ *∗*(*x*_*r*2_^*G*^ − *x*_*r*3_^*G*^)).

## 8. Problem Solution

**Figure 3:**
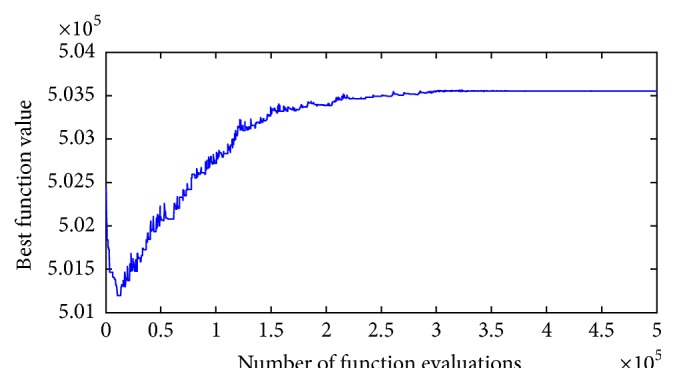
Convergence graph (best curve) of COMDE on the test problem.

The proposed GPM for the admission problem discussed in the previous sections has been tested. The experiments were carried out on an Intel Pentium core 2 due processor 2200 MHz and 2 GB RAM. COMDE algorithm is coded and realized in MATLAB. The best result in terms of the objective function value and the optimal decision variables is given in Table 3. 30 independent runs are performed and statistical results are provided including the best, median, mean, and worst results and the standard deviation is presented in Table 4. The convergence graph corresponding to the best objective function value *f*(*x*) of the best run of the case study against Total Number of Function Evolutions (TNFE) of the COMDE is shown in Figure 3.

In this goal programming model, all the goals for the enrollment part are considered with the same importance and are given equal weights of 1 in the objective function. The increase in the graduation rate is given higher priority than the number of the available jobs.

In the optimal solution, some of the goals are satisfied while some others are over- or underachieved:

- Goals numbers 3, 4, 5, 6, 7, and 8 for enrollment part and goals (19–24) for the graduation part are exactly satisfied.
- Goals numbers 1, 2, 9, and 16 for the enrollment part are underachieved.
- Goals numbers 10, 11, 12, 13, 14, and 15 for the enrollment part and goals (25–30) for the graduation part are overachieved.

So, we have the following:

- Exact satisfaction of the control for the education tracks, total number of enrollment students in relation to high school graduates, and number of population and student-to-faculty members for medical boys section.
- Underachievement of the enrollment rate increase, student-to-faculty members for medical girls section, and budget.
- Overachievement of student-to-faculty members for all specialties, boys and girls, except for student-to-faculty members for medical specialties, boys and girls sections.
- Exact satisfaction of the increase in the number of graduated students and overachievement of the available number of jobs.

From Table 4, it can be seen that the best results obtained by COMDE are the optimal feasible solution as the constraints are satisfied. Besides, from Table 5, COMDE is unable to reach the best solution consistently in all runs as the problem is very difficult as discussed previously. However, the median, mean, and worst solutions obtained by COMDE are not far from the best with small standard deviations which prove COMDE is robust technique. Moreover, convergence behavior is another important factor to be considered in solving optimization problems using evolutionary algorithms. From Figure 3, it can be deduced that the optimal solution can be reached using around 70% of total number of function evaluations which shows that COMDE is an efficient algorithm with rapid convergence speed. Based on the above results and analysis, it can be concluded that COMDE algorithm has a remarkable ability to solve considered nonlinear integer GP problems with a perfect performance in terms of high quality solution, rapid convergence speed, efficiency, and robustness.

## 9. Conclusions and Points for Future Researches

The national strategic plan for the admission capacity problem in higher education can be satisfactorily designed using a general nonlinear integer goal programming model. The model is formulated in a general form to satisfy the main objectives stated in the development plan of a country or an institution. The main objectives for the model are to cope with the increasing demand for higher education in the country and to satisfy the job market requirements, fair student satisfaction, and control over the education tracks (medical, science and engineering, and arts) under the limitation of available resources. The procedure of solution is a stepwise one; the mode is initially formulated for the first year of the plan. The obtained results are entered as input for the second year, and so on, till reaching the last year of the plan.

A recent novel differential evolution algorithm is used to find the optimum solution for many scenarios representing different priorities for the problem goals.

As future researches, it is proposed to consider the following points:

- To apply the same model for different countries taking into consideration the special goals stated in their strategic development plans.
- To formulate a multiobjective mathematical programming model for the same problem and consider different utility functions and paretooptimal solutions.
- To design a complete decision support system with user-friendly interfaces to facilitate the task for the decision-makers.

## Figures and Tables

**Algorithm 1:**
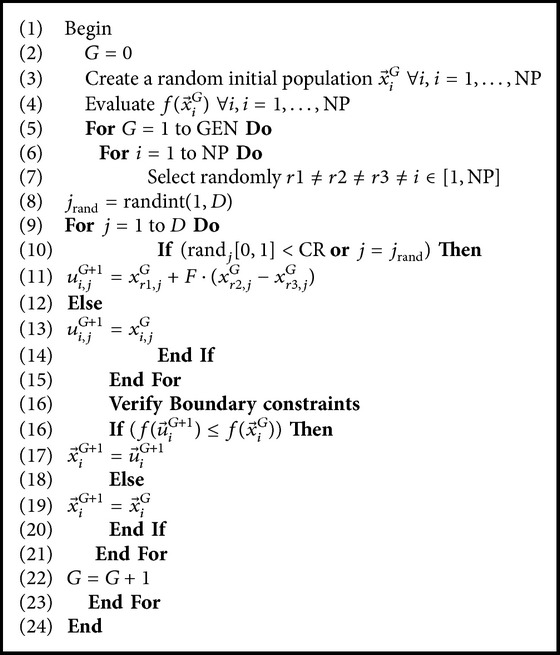
Description of standard DE algorithm.

**Algorithm 2:**
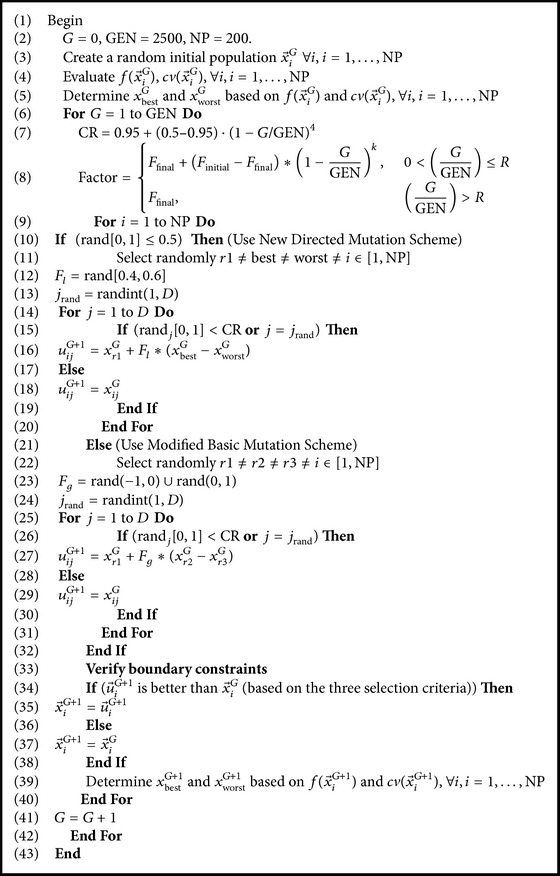
Description of COMDE algorithm.

**Table 1:** Problem attributes and their values.

Attributes	Values
*y* = year of the plan	*y* = 1 for the first year of the next plan (2015), 2 for the second, 3 for the third, 4 for the fourth, and 5 for the last*y* = 0 for the last year in the previous plan (current year, 2014), −1 for 2013, and so on

*u* = university	*u* = a university, *u* ∈ *U*, the set of all universities in the country

*i* = status	*E* stands for “enrolled” and *G* for “graduated”

*j* = gender	*b* stands for boys section and *g* for girls section

*k* = education program	*m* = a college in the medicine specialty, *m* ∈ *M*, the set of all colleges in the medicine specialty *s* = a college in the science and engineering specialty, *s* ∈ *S*, the set of all colleges in the science and engineering specialty *a* = a college in the arts specialty, *a* ∈ *A*, the set of all colleges in the arts specialty, and *T* is the total number in all specialties *M*, *S*, and *A*

**Table 2:** Relevant data related to the Kingdom of Saudi Arabia.

Sym.	Meaning	Value
*x*_*E*,*b*,*T*_^0,*U*^	Total number of boys enrolled in Saudi Arabia in all universities in 2014 (last year)	205,362
*x*_*E*,*g*,*T*_^0,*U*^	Total number of girls enrolled in Saudi Arabia in all universities in 2014 (last year)	190,608
*N*_*C*_^0^	Population of Saudi Arabia in the age of 18–23 years in 2014	1,036,700
*N*_*H*_^0^	High school graduates in the kingdom in 2014	380,050
*N*_*b*_^0^	Number of boys for bachelor scholarships abroad in 2014	22,644
*N*_*g*_^0^	Number of girls for bachelor scholarships abroad in 2014	8,477
*F*_*b*,*M*_^1,*U*^	Current number of faculties in all universities (boys section, medical specialty)	7,425
*F*_*g*,*M*_^1,*U*^	Current number of faculties in all universities (girls section, medical specialty)	4,433
*F*_*b*,*S*_^1,*U*^	Current number of faculties in all universities (boys section, science and engineering specialty)	19,346
*F*_*g*,*S*_^1,*U*^	Current number of faculties in all universities (girls section, science and engineering specialty)	8,658
*F*_*b*,*A*_^1,*U*^	Current number of faculties in all universities (boys section, arts specialty)	9,431
*F*_*g*,*A*_^1,*U*^	Current number of faculties in all universities (girls section, arts specialty)	9,776
*F*_*b*,*T*_^1,*U*^	Current number of faculties in all universities (boys section, all specialties)	37,245
*F*_*g*,*T*_^1,*U*^	Current number of faculties in all universities (girls section, all specialties)	23,405
*B* ^1,*U*^	Total budget for the current year in all universities in the kingdom	4,778.5 million
*c* ^1,*U*^	Average cost of one student at the kingdom level in the current year	56,250
*x*_*E*,*b*,*M*_^−5,*U*^	Number of enrolled boys in the kingdom in 2009 (medicine)	8,518
*x*_*G*,*b*,*M*_^0,*U*^	Number of graduated boys in the kingdom in 2014 (medicine)	3,191
*x*_*E*,*b*,*S*_^−4,*U*^	Number of enrolled boys in the kingdom in 2010 (science and engineering)	93,807
*x*_*G*,*b*,*S*_^0,*U*^	Number of graduated boys in the kingdom in 2014 (science and engineering)	18,103
*x*_*E*,*b*,*A*_^−3,*U*^	Number of enrolled boys in the kingdom in 2011 (arts)	34,301
*x*_*G*,*b*,*A*_^0,*U*^	Number of graduated boys in the kingdom in 2014 (arts)	13,851
*x*_*E*,*g*,*M*_^−5,*U*^	Number of enrolled girls in the kingdom in 2009 (medicine)	7,114
*x*_*G*,*g*,*M*_^0,*U*^	Number of graduated girls in the kingdom in 2014 (medicine)	3,456
*x*_*E*,*g*,*S*_^−4,*U*^	Number of enrolled girls in the kingdom in 2010 (science and engineering)	92,867
*x*_*G*,*g*,*S*_^0,*U*^	Number of graduated girls in the kingdom in 2014 (science and engineering)	22,871
*x*_*E*,*g*,*A*_^−3,*U*^	Number of enrolled girls in the kingdom in 2011 (arts)	38,993
*x*_*G*,*g*,*A*_^0,*U*^	Number of graduated girls in the kingdom in 2014 (arts)	34,797

**Table 3:** Parameter settings.

Control parameter	Actual values
Population size (NP)	200
Maximum generations (GEN)	2500
Crossover rate (CR)	CR = 0.95 + (0.5–0.95) · (1 − *G*/GEN)^4^
Local scaling factor (*F*_*l*_)	Uniform random number [0.4, 0.6]
Global scaling factor (*F*_*g*_)	Uniform random number (−1,0)∪(0,1)

**Table 4:** Optimal values of nonzero design variables for the case study.

Number	Decision variable	Optimal solution
1	*x*_*E*,*b*,*T*_^1,*U*^	136,125
2	*x*_*E*,*g*,*T*_^1,*U*^	72,903
3	*x*_*E*,*b*,*M*_^1,*U*^	7,425
4	*x*_*E*,*b*,*S*_^1,*U*^	74,250
5	*x*_*E*,*g*,*M*_^1,*U*^	3,977
6	*x*_*E*,*g*,*S*_^1,*U*^	39,765
7	*x*_*E*,*b*,*A*_^1,*U*^	54,450
8	*x*_*E*,*g*,*A*_^1,*U*^	29,161
9	*x*_*G*,*b*,*M*_^1,*U*^	7,241
10	*x*_*G*,*b*,*S*_^1,*U*^	79,736
11	*x*_*G*,*b*,*A*_^1,*U*^	29,156
12	*x*_*G*,*g*,*M*_^1,*U*^	6,047
13	*x*_*G*,*g*,*S*_^1,*U*^	78,937
14	*x*_*G*,*g*,*A*_^1,*U*^	33,145
	*z* = sum of nonzero deviation variables	503,555

**Table 5:** The statistical results of COMDE on the test problem.

	Best	Median	Mean	Worst	Std.
Test problem	503,555	503,567	503,573	503,595	13.0026
